# Experimental Investigation of the Friction Stir Weldability of AA8006 with Zirconia Particle Reinforcement and Optimized Process Parameters

**DOI:** 10.3390/ma14112782

**Published:** 2021-05-24

**Authors:** Thanikodi Sathish, Abdul Razak R. Kaladgi, V. Mohanavel, K. Arul, Asif Afzal, Abdul Aabid, Muneer Baig, Bahaa Saleh

**Affiliations:** 1 Department of Mechanical Engineering, Saveetha School of Engineering, Saveetha Institute of Medical and Technical Sciences (SIMATS), Chennai 602105, Tamil Nadu, India; 2 Department of Mechanical Engineering, P.A. College of Engineering (Affiliated to Visvesvaraya Technological University, Belagavi), Mangaluru 574153, Karnataka, India; arkmech9@gmail.com; 3 Centre for Materials Engineering and Regenerative Medicine, Bharath Institute of Higher Education and Research, Chennai 600073, Tamilnadu, India; 4 Department of Mechanical Engineering, Jeppiaar SRR Engineering College, Chennai 603103, Tamilnadu, India; arulroll7@gmail.com; 5 Engineering Management Department, College of Engineering, Prince Sultan University, P.O. Box 66833, Riyadh 11586, Saudi Arabia; aaabid@psu.edu.sa (A.A.); mbaig@psu.edu.sa (M.B.); 6 Mechanical Engineering Department, College of Engineering, Taif University, P.O. Box 11099, Taif 21944, Saudi Arabia; b.saleh@tu.edu.sa

**Keywords:** friction stir welding, AA8006 alloy, zirconia, SEM analysis, 3D profilometry and wrought alloy

## Abstract

A lightweight, highly corrosive resistant, and high-strength wrought alloy in the aluminum family is the Aluminium 8006 alloy. The AA8006 alloy can be formed, welded, and adhesively bonded. However, the recommended welding methods such as laser, TIG (Tungsten Inert Gas welding), and ultrasonic are more costly. This investigation aims to reduce the cost of welding without compromising joint quality by means of friction stir welding. The aluminum alloy-friendly reinforcement agent zirconia is utilized as particles during the weld to improve the performance of the newly identified material AA8006 alloy in friction stir welding (FSW). The objectives of this research are to identify the level of process parameters for the friction stir welding of AA8006 to reduce the variability by the trial-and-error experimental method, thereby reducing the number of samples needing to be characterized to optimize the process parameters. To enhance the quality of the weld, the friction stir processing concept will be adapted with zirconia reinforcement during welding. The friction stir-processed samples were investigated regarding their mechanical properties such as tensile strength and Vickers microhardness. The welded samples were included in the corrosion testing to ensure that no foreign corrosive elements were included during the welding. The quality of the weld was investigated in terms of its surface morphology, including aspects such as the dispersion of reinforced particles on the welded area, the incorporation of foreign elements during the weld, micro defects or damage, and other notable changes through scanning electron microscopy analysis. The process of 3D profilometry was employed to perform optical microscopy investigation on the specimens inspected to ensure their surface quality and finish. Based on the outcomes, the optimal process parameters are suggested. Future directions for further investigation are highlighted.

## 1. Introduction

The solid-state welding method of Friction Stir Welding (FSW) is a newly modified process. This process is effectively utilized for altering surface structures and improving materials’ physical properties. The term friction stir processing (FSP) refers to surface modification through reinforcements in addition to friction stir welding [1,2,3,4,5,6,7,8,9,10]. This research investigates the effects of the novel reinforcement of zirconia particles on the welding zone of the friction stir welding process of the AA8006 work material. Various research findings support the continuous improvement of this process and describe the effective welding of similar and dissimilar metals by friction stir welding. The term friction stir processing (FSP) refers to surface modification by means of reinforcement in addition to friction stir welding. Abhishek et al. [1] analyzed the joining of the AA 6061 alloy by FSP and utilized silicon carbide-graphite as a reinforcement to form a hybrid composite weld material, finding that the strength of the weld improved. The uniform dispersion of the reinforcement agent was ensured by microstructural investigation. It was reported that the stirring speed is a major influencing factor and is responsible for modifying the mechanical properties of hybrid composite welds. The use of a high rotational speed of 2200 rpm at 25 mm/min for traverse speed forms a strong weld. Kan et al. [2] recommends the use of FSP, as it develops a defect-free weld and can form a composite weld material at joints with excellent hardness and wear properties. It was found that though the tool pin was considered durable, it suffers from wear over time and such wear is unavoidable due to friction and heat while stirring. Such wear will increase the tool and welding cost. Apart from the use of aluminum metal matrix composite weld metal on aluminum welding joints, Palanivel et al. [3] introduced a hybrid Aluminium Matrix Composites (AMC) (reinforced with titanium boride boron nitride) composite weld material. This is titanium boride and boron nitride introduced as a tribo film form in the FSP of aluminum alloy 6082 on a welding zone to form a hybrid AMC composite weld material. It was found that TiB_2_ particles were broken and boron nitride particles were not broken on the weld material. This method can improve the wear resistance. Boron nitride reduces the debris in the wear caused by the iron content and could possibly also reduce the counter face wear. Harikrishna Rana and Vishvesh Badheka et al. [4] welded AA7075 work materials with boron carbide reinforcement using FSP and created an aluminum matrix composite weld material at the joint. The maximum stirring speed and frequently changes the traverse speed, producing more heat, it can be reduced the wear resistance. As tool pin profile is a major influencing factor, Saravanan et al. [5,6] optimized the tool pin profile to improve the welding quality. They utilized the FSW of the dissimilar aluminum alloys of AA 2014 and AA 7075. In their investigation, they made only three joints by varying the stirring speed of the tool pin, altering the fixed material’s situation at withdrawing as well as progressing sides, and achieved a maximum rigidity of 207 MPa. Pandiyarajanet al. [7] investigated the joining of AA6061 plates by FSP with zirconia reinforcement and ensured that the reinforcements showed a uniform distribution of ZrO_2_ on the weld material through Scanning Electron Microscopy (SEM) analysis; the hardness at the joints significantly improved. Sivaraman et al. [8] also investigated the development of the dissimilar metals AA 2014 and AA 7075 through friction stir welding. They altered the rotational speed and material position to obtain a better impact and tensile strength. Ramamoorthi et al. [9] analyzed the friction stir welding of the dissimilar metals, AA5086 and AA6063, by varying the axial force, and reported that axial force strongly influenc was the mechanical properties of the welded joints. Fakkir Mohamed et al. [10] also investigated the FSW of the dissimilar metals AA5083 and AA6061 by varying three important parameters: load, feed, and speed. The speed is a more influential parameter than the other two in producing a high standard of welding. Ref. [11] achieved a high UTS of 920 MPa with 37% elongation in the FSW of Al0.3CoCrCu0.3FeNi high-entropy alloy through the microstructure of the stir zone, which was partially re-crystallized and fine grained. This investigation utilized a novel approach toAA8006 welding by means of FSP with zirconia particle reinforcement. The aluminum alloy AA8006 is usually used in costly welding methods such as ultrasonic welding, laser welding, and Tungsten Inert Gas (TIG) welding. This investigation aims to reduce the cost of welding AA8006 without compromising the weld quality by FSP. Hence, this investigation is completely unique, as we attempt to use new techniques to increase the applications of AA8006. It is important to investigate this material because, compared to other aluminum alloys, the 8006 alloy possesses superior corrosion resistance and high strength. It is a category of wrought alloys that can be treated thermo-mechanically to improve their strength.

## 2. Materials and Methods

### 2.1. Base Material

Aluminium 8006 alloy is a high-strength (elastic modulus 70MPa) and highly corrosive resistant alloy. It is often referred as a wrought alloy. The AA 8006 was named a medium-strength alloy; it is used in many different applications, such as in making containers, heat exchanger units, tin boxes, and cable covers, etc. This material has a great formability, with a good mechanical strength. The constituents of the AA8006 alloy are presented in Table 1.

### 2.2. Reinforcement Agent

In this study, the AA8006 aluminum alloy is taken as a base alloy and the packing of reinforced material such as zirconia and the fabrication can be carried out by the use of FSP technology. The reinforcement of zirconium dioxide is preferred, as it possesses an excellent stability against mechanical stresses and is resistant to cracking and crack growth. Apart from its high flexural strength (up to 1000 MPa), it has a fracture toughness of 10 MPa M^1/2^ at 20 °C, a tensile strength of 30 Mpa, and an elastic modulus of up to 250 GPa. Zirconia is an inorganic metal oxide that can act as a successful reinforcement material for the property enhancement of aluminum alloys. It has proven inclusion effects in many aluminum metal matrix composites as well as in FSP. Zirconia is mainly utilized in the reinforcement of ceramic materials; it is mainly applied in the production of abrasive parts, dental applications, and fuel cells [7].

### 2.3. Experimentation

As described above, the number of process variables was reduced by trial-and-error experiments, because to our best knowledge no previous work on AA8006 has been reported so far in the literature. Therefore, the weldability was justified by trial-and-error experimentations. To enhance the quality of the weld, the friction stirs processing concept was adapted with zirconia reinforcement at the weld. We utilized 6 mm-thick, 150 × 75 mm rectangular-shaped samples plates for FSP. The American Society for Testing Materials ASTM D638 standard was adhered to and a computer-controlled 100 KN capacity Universal Testing Machine (model: SICMUTM-01, manufacturer: Shambhavi, city: Navi, state: Mumbai) was employed in this investigation [12,13,14,15].

FSP is a novel route developed from the origin of the FSW process, the reinforcement tactic used to modify the surface microstructure of the material while in FSP. Table 2 shows the different ranges of tool pin speed, traverse speed, tool downward force, and tool tilt angle. Five samples in each experimental group (stirring speed setting) were investigated and the average of their values were considered for evaluation and in drawing conclusions.

**Figure 1:**
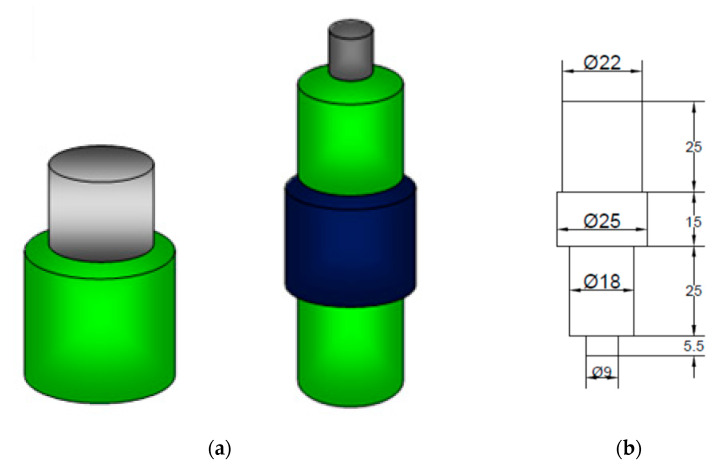

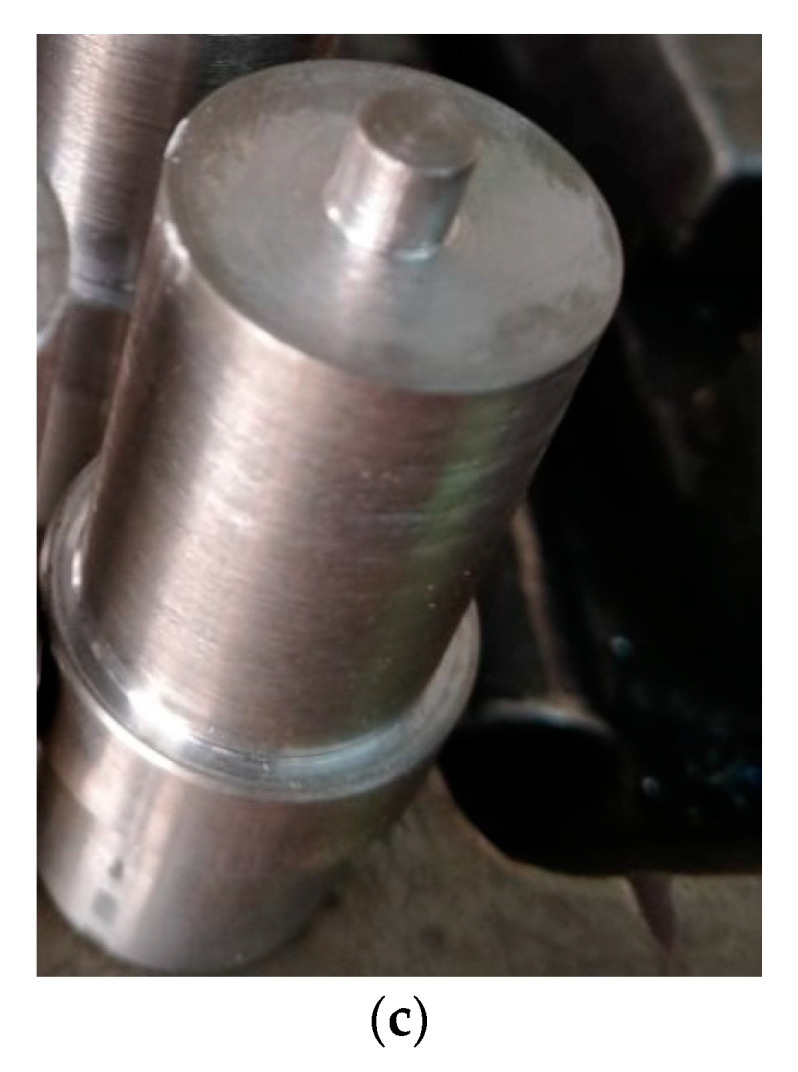
FSP tool: (**a**) compacting and FSP tool shape feature; (**b**) dimensional feature of FSP tool; (**c**) fabricated FSP tool.

The designed tool shoulder and straight cylindrical tool pin are depicted in Figure 1. Figure 1a shows a conceptual design of the FSP tool and compacting tool. Its dimensional features are shown in Figure 1b and the actual fabricated tool is depicted in Figure 1c. Tools are utilized in a Computerized Numerical Control (CNC), (model: JV 30, manufacturer: Lakshmi Machine works, city: Coimbatore, state: Tamil Nadu) machine and the tool is made out of high-carbon high chromium steel.

**Figure 2:**
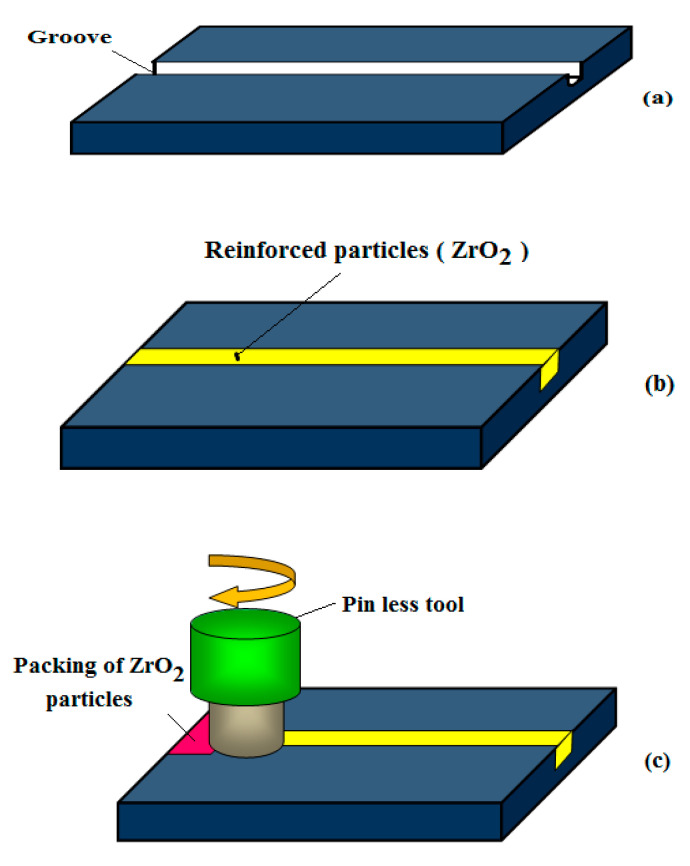

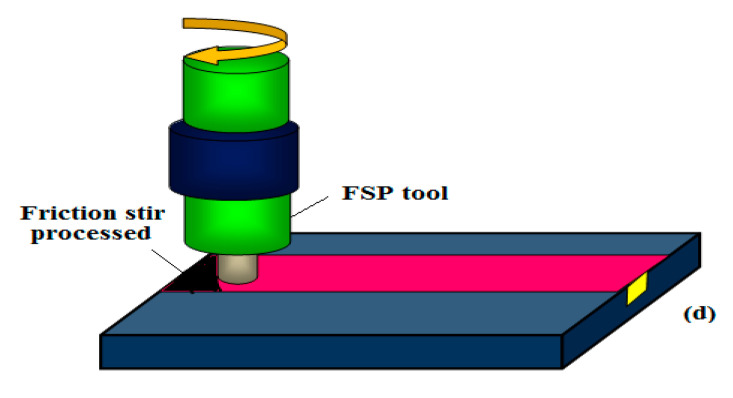
FSP process procedure: (**a**) square groove on the base material; (**b**) reinforced particles filling in the groove; (**c**) compaction process using a pinless tool; and (**d**) FSP process.

The base material of the AA 8006 has the dimensions of 200 × 100 × 10 mm. The top surface of the samples has a square groove depth of 5 mm and width of 3 mm, which are cut using the wire EDM process, as shown in Figure 2a. The next stage of the reinforced particles of zirconium dioxide is filling in the groove on the base material surface and tightly compressing them, as shown in Figure 2b.

The packing of reinforced particles in the groove was achieved with the help of a pinless tool, as shown in Figure 2c. A CNC vertical milling machine was used to rotate the pinless tool, pressing the particles tightly and closing the groove without the reinforced particles escaping. The FSP process was carried out finally with the assistance of a straight cylindrical pin tool, as shown in Figure 2d. The FSP process mixed the zirconium dioxide particles into base materials with a different tool rotational speed; due to the heat generation, the particles are mixed well into base materials [15]. 

### 2.4. Characterization for Tensile Strength of Welded Samples

**Figure 3:**
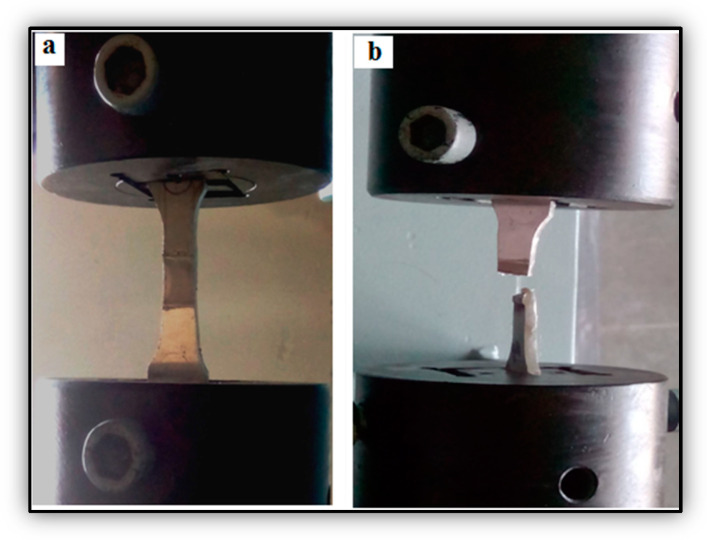
Image of tensile test (**a**) before fracture, (**b**) after fracture.

The upgrading of the surface structure by FSP increased the strength of the specimens to a maximum level. The four samples were tested to see if they met the ASTM E8M-04 standard; the 40-ton capacity of Universal Testing Machine (UTM) was used, Figure 3 was illustrates to employed for conducting tensile strength testing on the samples. The strain rate was measured with the aid of an extensometer [16,17,18]. The four samples were tested at 1100 rpm. The FSP showed that, the sample tensile strength was moderately increased, proving that a higher tool rotational speed drastically modified the surface structure. The thickness of the specimen was 3 mm. The sample width and length were 25 and 240 mm, respectively. The specimen was loaded vertically along the loading direction and gripped. A uniform displacement of 0.02 cm per min was applied. The dimensions of the broken area were measured with a vernier caliper and the cross section was calculated. 

### 2.5. Characterization for Hardness of Welded Samples

In this section, the microhardness of the specimens at various locations from the welded zone is analyzed. We examined how the microhardness varied from the center of the weld with respect to a uniform distance to the extreme edges of the plate. The FSP zone was the center of the weld, the thermo-mechanically affected zone was the portion that suffered heat and mechanical force over the tool head, the heat-affected zone was next to the welded area that suffered heat generated during the FSP, and the unaffected zone was next to the heat-affected zone and the edge of the plates. The Vickers microhardness tester (model: BHT 1000, manufacturer: Acme Engineers, city: Dhayari, state: Pune) was employed for this investigation. The preparation of the samples included them being finished and polished using a fine 800 grit emery sheet. The hardness measurement was taken in all the zones mentioned. Different test conditions were applied for a load of 400 g and maintained for 20 s; the indentation was analyzed in all samples and the microhardness values were recorded [19].

### 2.6. Characterization for Corrosion on Welded Samples

**Figure 4:**
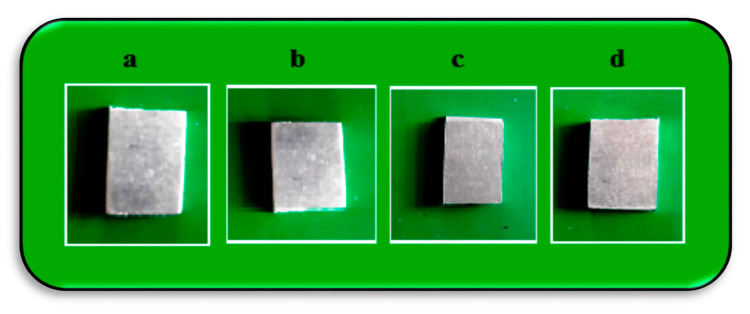
Corrosion tested samples of (**a**) Expt. 1, (**b**) Expt. 2, (**c**) Expt. 3, and (**d**) Expt. 4.

The immersion corrosion test was conducted effectively with the specified ASTM standards G31-72; samples were prepared with dimensions of 20 × 15 × 6 mm from each specimen (see Figure 4) at the FSP zone. The surface of the specimens was polished using a 800 grit emery and cleaned by kerosene medium. The mass loss of all the specimens was measured directly by a digital weight balance; the samples were weighted before and after immersion. 

The corrosion characteristics of the samples were analyzed by way of an immersion test. The corrosion medium was prepared at a 3.5% NaCl level and all the samples were immersed individually in the corrosion solution. The immersion time period maintained for the test was 48, 72, 96, and 120 hrs. After finishing the immersion time period, the samples were taken out, allowed to dry, then weighted [20]. 

The formula used for finding the corrosion rate of the specimens was:

(1) $$\mathrm{Corrosion}\;\mathrm{rate}\;\left(\mathrm{mm}/\mathrm{yr}\right)=\frac{k\;\times \Delta W\;}{a_{c}\times {\;t}_{e}\times \;\rho },$$

where k = 8.76 × 10^4^,t_e_ = time of exposure (hours), a_c_ = cross sectional area of the specimen (cm^2^), ∆W = reduction in specimen weight (g), *ρ* = mass density of the material (g/cm^3^).

## 3. Results and Discussion

Different speeds (800, 900, 1000, and 1100 rpm) were used to produce the FSP samples for the tensile test. The specimens were tested, and the tensile strength of each specimen is shown in Table 3.

### 3.1. Tensile Strength of Friction Stir Processed Samples

**Figure 5:**
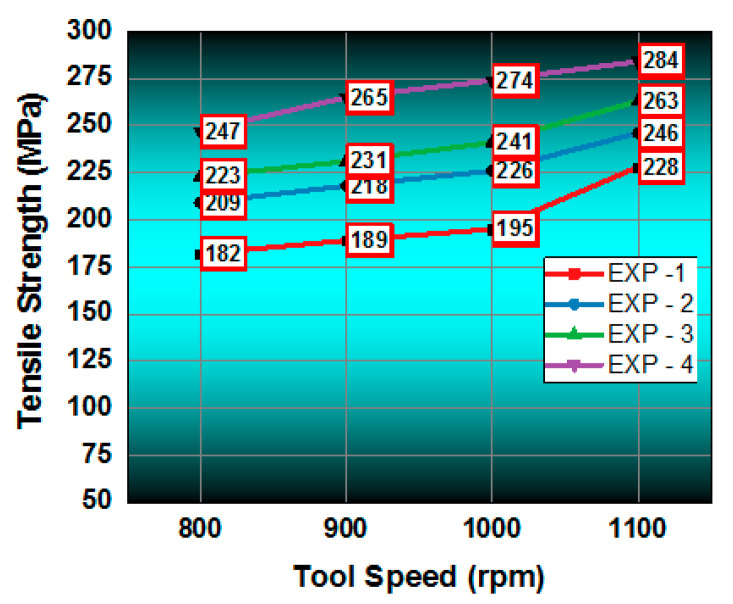
Average tensile strength of the welded joints at various stirring speeds.

From the tensile test, Figure 5 shows the connection between the stirring speed (tool rotational) and the respective tensile strength of the weld produced on the sample. The four samples and their tensile strengths were analyzed; the maximum tensile strength of 284 MPa was reached for specimens which experienced a stirring speed of 1100 rpm. The increase in stirring speed increased the strength of the welding, helping it to withstand tensile loads, and vice versa. High speeds induced a fine grain structure in the FSP zone. Table 3 shows the details of the process parameters employed in each experiment. Four samples were prepared and tested.

**Figure 6:**
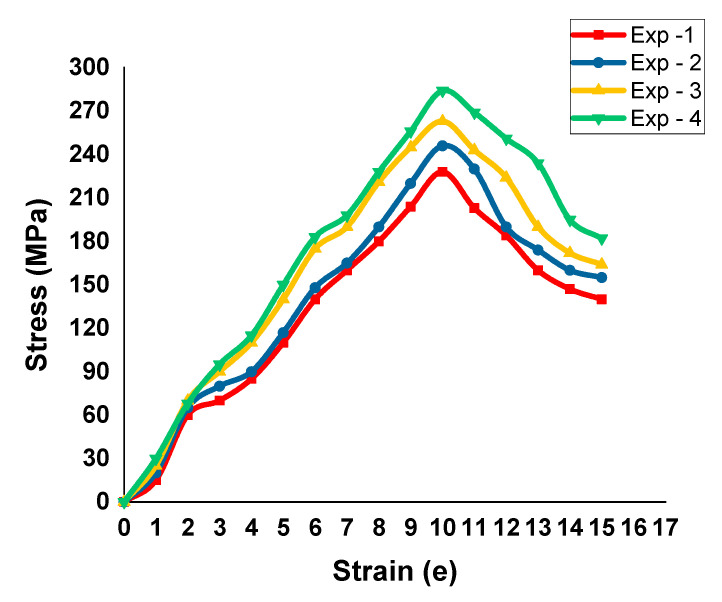
Stress strain curves of the different specimens.

Figure 6 presents the stress–strain curve of the tensile test specimens. From the four tool speeds, the maximum tool speed (1100 rpm) was produced the best tensile strength in the specimens. In the four tensile tests, the maximum tensile strength was recorded as 284 MPa and the minimum tensile strength was registered as 228 MPa.

### 3.2. Hardness on Friction Stir Processed Samples

Table 4 presents the microhardness values for the various zones of the FSP using different tool speeds. Among the four speeds used, using a tool speed of 1100 rpm produced a high microhardness value of 148 Vickers Hardness Number (VHN) in the FSP zone. In the thermo-mechanically affected zone, it was 145 VHN; in the heat-affected zone, it was 142 VHN; and in the unaffected zone, it was 84 VHN.

**Figure 7:**
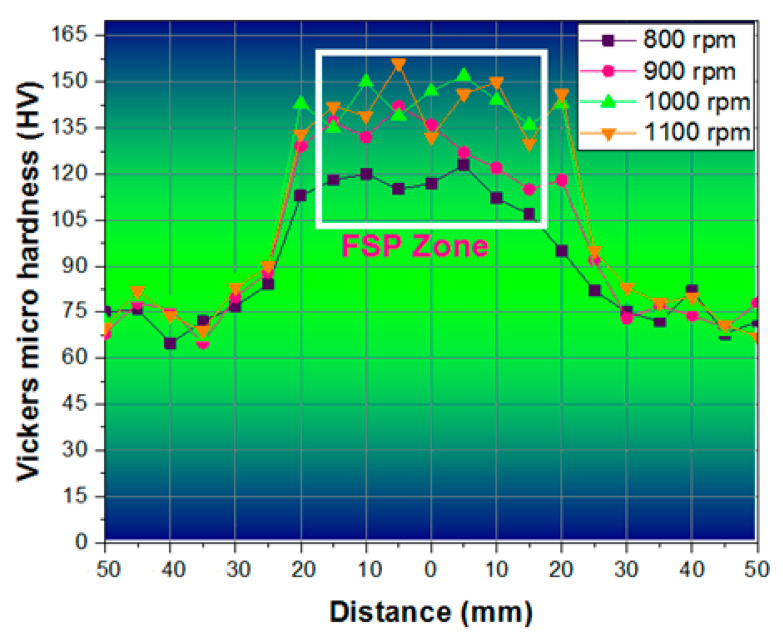
Vickers microhardness test graph (distance vs. microhardness).

The influence of FSP on the samples in terms of microhardness variation from the center of the weld to the extreme edges of the plate was investigated and presented in graphical form in Figure 7. The hardness was measured in the FSP zone (that is, the center of the weld), the thermo-mechanically affected zone (that is, the portion that suffered heat and mechanical force over the tool head), the heat-affected zone (next to the welded area that suffered heat generated during the FSP), and the unaffected zone (next to the heat-affected zone to edge of the plates). From the welding zone to the edge of the specimens, the length was divided into 10 equal distances on both sides (5 mm per division) and measurements were carried out.

The maximum hardness values were found in the friction stir processing zone. In the base (parent) material zone, the value of hardness was obtained in the range of 60 to 80 HV, but in the FSP zone the range was from 115 to 155 HV [21]. During FSP, high tool speeds broke the hard grains to form fine grains in the structure at the zone of stirring, thus improving the hardness. An excellent hardness value of 156 HV was found in the zirconia loaded on the groove between the sample edges (the FSP stirring zone) with regard to the tool rotational speed (1100 rpm). The zirconium dioxide intensified the hardness value in the FSP zone [22].

### 3.3. Corrosion Test Results of Friction Stir Processed Samples

Immersion corrosion testing was carried out at different time periods with 3.5% of NaCl corrosion medium, as illustrated in Table 5. The tool rotational speed and corrosion rate are shown in Table 4. 

**Figure 8:**
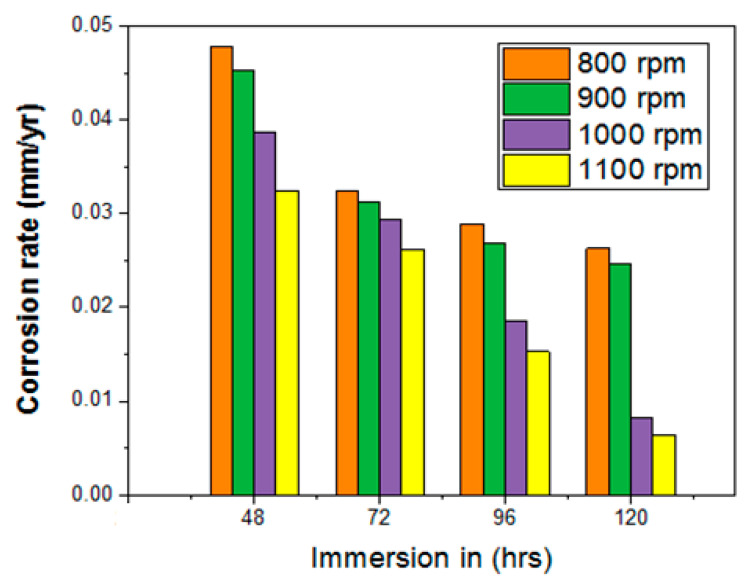
Corrosion test graph, immersion vs. corrosion rate.

The highest corrosion rate was obtained at the start of the test. Further increasing the time period caused the corrosion rate of the all samples to decrease consistently. The lowest corrosion rate of 0.0064 mm/yr was attained when a higher stirring speed (tool rotational) of 1100 rpm was influenced. Figure 8 shows the relationship between the immersion and corrosion rate in relation to the stirring speed (tool rotational). Hence, it is understood that an increase in stirring speed reduces the corrosion rate significantly. Across the whole immersion time period, the sample for which a 1100 rpm tool rotational speed was used attained an excellent corrosion resistance. Microstructure alteration increases the corrosion resistance, suggesting that tool speed is the major factor in FSP [23,24,25,26,27,28].

### 3.4. Results of Optical Microscopic Analysis on Friction Stir Processed Samples

**Figure 9:**
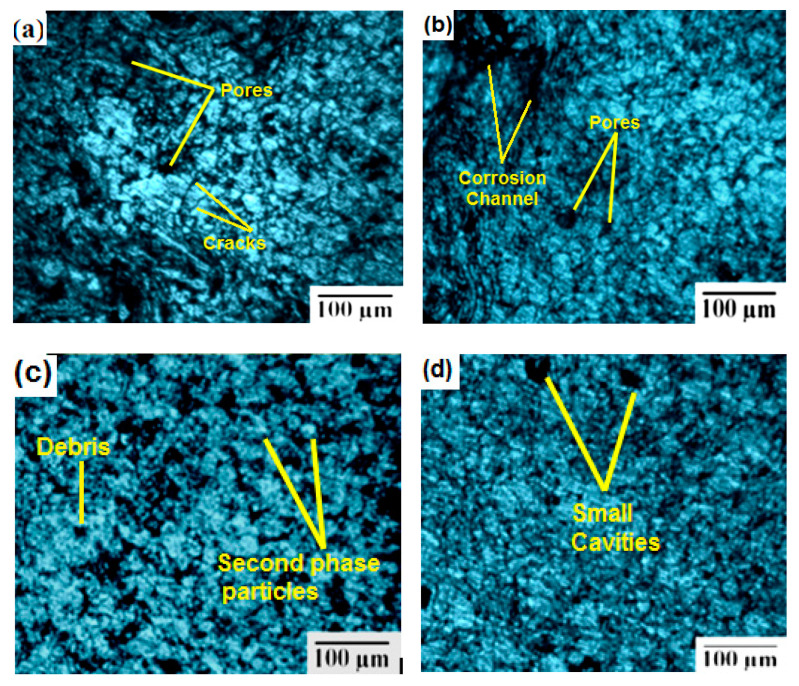
Optical microscopic image of corrosion test specimen: (**a**) Expt. 1, (**b**) Expt. 2, (**c**) Expt. 3, and (**d**) Expt. 4.

Optical microscopic images of the immersion corrosion test specimens were obtained. Figure 9 presents the four SEM images of the after-corrosion test specimens. These images show that the use of different tool speeds causes various defects on the corroded specimens. Defects of small holes due to variation in the melting of the grains are shown in Figure 9a–d. The images in Figure 9a,b show that the corrosion takes place evenly and that the zirconium dioxide particles are mixed well and appear on the surface randomly. In Figure 9c, it can be seen that the zirconium oxide particles are mixed uniformly and only a small amount of corrosion takes place. From Figure 9d, it can be seen that surface alteration can be performed effectively and very little corrosion occurs in the FSP zone. Different defects were identified in the images, such as pores, cracks, corrosion channels, second phase particles, small cavities, and debris. The corrosion varied based on the tool speed. A high tool speed offered low corrosion defects.

### 3.5. Surface Morphological Investigation on Samples with 2D Profilometry Images

**Figure 10:**
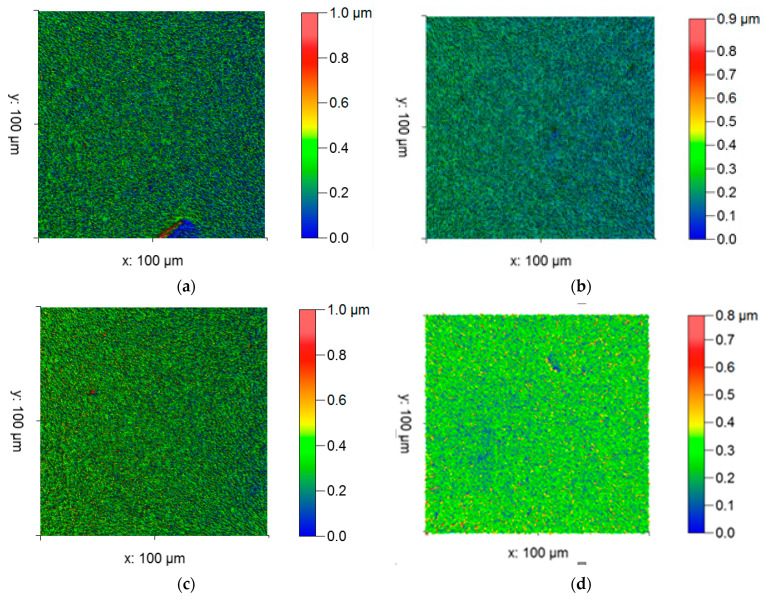
2D laser profilometry of the corrosion test. (**a**) Expt. 1, (**b**) Expt. 2, (**c**) Expt. 3, and (**d**) Expt. 4.

In Figure 10a, it can be seen that the lack of stirring action in the bottom area causes a higher corrosion rate compared to the other samples. Figure 10b shows that a reasonable corrosion rate was attained due to low rotational speed, due to lack of grain modification in the FSP and it shown in a blue color. Figure 10c,d show that the corrosion takes place only in small amounts due to the high speed involved; the blue color is lightly shown in the image.

### 3.6. Surface Morphological Investigation on Samples with 3D Profilometry Images

**Figure 11:**
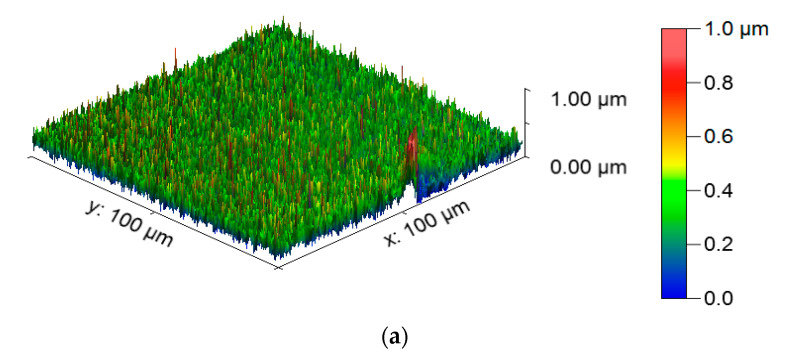

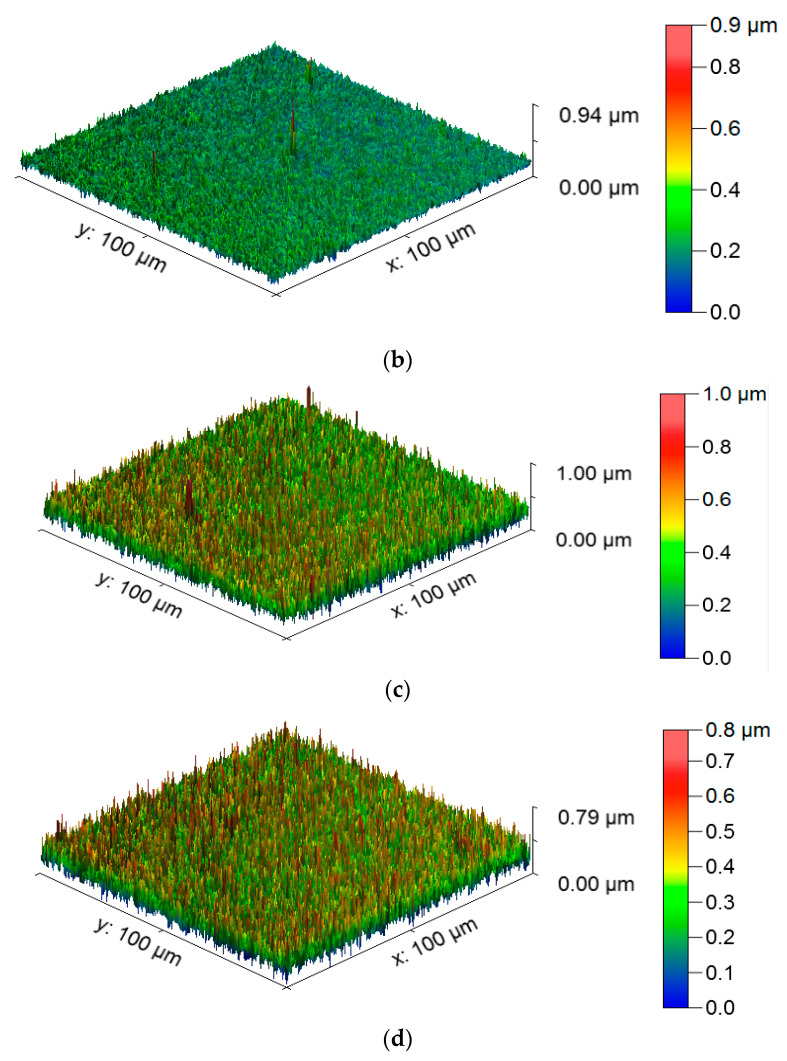
3D laser profilometry of the corrosion test: (**a**) Expt. 1, (**b**) Expt. 2, (**c**) Expt. 3, and (**d**) Expt. 4.

The SEM images (SEM; HITACHI, tested at AC Tech., Anna University, Chennai, India) were converted into 3D profilometry images of the corrosion test specimens; these are shown in Figure 10. The color scale bar indicates the blue, green, brown, and red colors for identifying the corrosion rate. In Figure 11a, the blue color shown in the bottom area shows that a large amount of corrosion took place. The small amount of blue color in Figure 11b denotes that the area was moderately corroded. Figure 11c,d shows a little blue color, meaning a small amount of corrosion took place. From all the images, it can be seen that a high tool rotational speed decreased the corrosion rate and increased the corrosion resistance due to the sufficient alteration in the grain structure.

## 4. Conclusions

The novel approach of friction stir processing for AA8006 with zirconium dioxide (zirconia) was carried out using a straight cylindrical tool. The investigations of the tensile strength corrosion resistance and the Vickers microhardness were conducted and the values were measured. The outcomes of this research are as follows:

- In the tensile test, all four specimens were tested, and all the specimens’ tensile strength increased with the increase in the stirring (tool rotational) speed. The maximum tensile strength obtained was 284 MPa with a stirring speed of 1100 rpm. This clearly confirms that an increased speed and high temperature modify the grains in the surface structure.
- The hardness development in the FSP samples revealed an improved Vickers microhardness of 156 HV at the friction stir processing zone for a higher stirring speed of 1100 rpm. The investigation results show that there was an increase in the Vickers microhardness at the friction stir processing zone with an increase in the stirring speed. The microhardness of the base materials also improved a little.
- The investigation of corrosion resistance in the friction stir processed samples revealed that the corrosion resistance could be altered by altering the stirring speed. The corrosion rate increased then gradually decreased when the length of immersion increased. The minimum corrosion rate achieved was 0.0064 mm/yr with a 1100 rpm stirring speed.
- OM and SEM images of the 2D and 3D profilometry were taken to see whether the corrosion resistance of the all specimens was improved by the friction stir processing, It was found that using a lower stirring speed causes small holes in the friction stir-processed specimens.
- Our findings are that the AA8006 material is friction stir weldable. The quality of the weld can be improved by friction stir processing. The rate of stirring decides the corrosion resistance, tensile strength, and microhardness in the welded zone. That is, the process can be controlled by altering the stirring speed to obtain the desired corrosion resistance, tensile strength, and microhardness.
- This investigation utilized the well-known reinforcement agent zirconia for friction stir processing. Our investigation will be extended with some other reinforcements. This investigation considered AA8006 alloy plates with a fixed thickness for FSP. The thickness will be altered and standardized for commercial use.

## Figures and Tables

**Table 1:** Constituents of the AA8006 alloy.

Name of the Constituent in AA8006 Alloy	Chemical Symbol	Percentage of Contribution
Zinc	Zn	0.75%
Magnesium	Mg	0.69%
Copper	Cu	0.27%
Silicon	Si	0.36%
Iron	Fe	1.60%
Manganese	Mn	0.58%
Aluminum	Al	95.60%
Other Elements Each	-	0.04%
Total Sum of Others	-	0.11%

**Table 2:** Description of theprocess parameters used for FSP.

Experimental. Index.	Stirring (Tool Pin Rotational) Speed (rpm)	Traverse Speed of Tool Head (mm per min)	Compressive Force on Tool (kN)	Tool Tilt Angle in Degree
1	800	40	3	2
2	900	40	3	2
3	1000	40	3	2
4	1100	40	3	2

**Table 3:** Friction stir processing parameters used.

Experimental. Index	Stirring (Tool Pin Rotational) Speed (rpm)	Traverse Speed of Tool Head (mm per min)	Compressive Force on Tool (kN)	Tool Tilt Angle in Degrees
1	800	40	3	2
2	900	40	3	2
3	1000	40	3	2
4	1100	40	3	2

**Table 4:** Microhardness for the various zones of the FSP using different tool speeds.

		Average Microhardness Values in Various Zones			
Expt. Index	Tool Pin Rotational Speed (rpm)	Unaffected Zone	Heat-Affected Zone	Thermo-Mechanically Affected Zone	FSP Zone
1	800	75	109	114	115
2	900	77	120	122	124
3	1000	80	133	138	141
4	1100	84	142	145	148

**Table 5:** Summary of the corrosion test.

Specimen No.	Tool Pin Rotational Speed (rpm)	Corrosion Rate (mm/yr)			
		48 h	72 h	96 h	120 h
1	800	0.0478	0.0324	0.0289	0.0263
2	900	0.0453	0.0312	0.0268	0.0247
3	1000	0.0387	0.0294	0.0186	0.00821
4	1100	0.0324	0.0262	0.0153	0.0064

## Data Availability

Raw data available from the corresponding authors upon request.

## Nomenclature

MPa	Mega Pascal
rpm	revolution per minute
Mm/min	millimeter per minute
FSP	Friction Stir Processing
FSW	Friction Stir Welding
SEM	Scanning Electron Microscopy
ON	Optical Microscopy
kN	kilo-Newton
t_e_	Time of Exposure
a_c_	cross sectional area
∆W	reduction in specimen weight
ρ	mass density
NaCl	sodium chloride
ASTM	American Society for Testing and Materials
G	Grams
UTM	Universal Testing Machine
CNC	Computer Numerical Control
ZrO_2_	zirconia (or) zirconium dioxide
TIG	Tungsten Inert Gas
AMC	aluminum metal matrix composite
TiB_2_	titanium diboride
BN	boron nitride
Al0.3CoCrCu0.3FeNi	a kind of high-entropy alloy
